# Supplementary figures and images for: Inhibitory circuit motifs in Drosophila larvae generate motor program diversity and variability
Source: PLoS Biol. 2025 Apr 21;23(4):e3003094. doi: 10.1371/journal.pbio.3003094 (PMC12088524; doi:10.1371/journal.pbio.3003094)

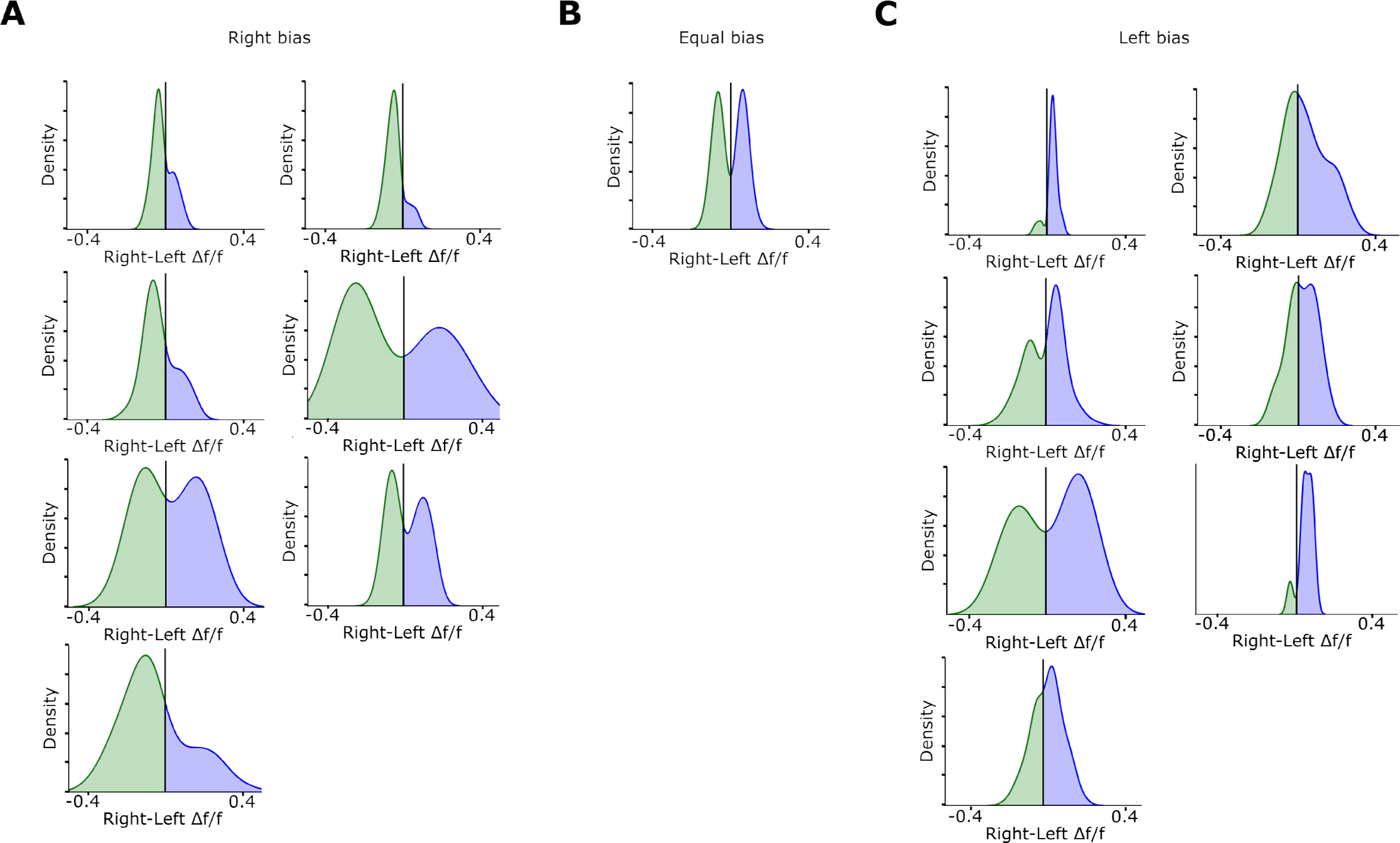

Supplement: S1 Fig — Kernel density plots showing all preparations that exhibited a fictive right (A), no (B), and left (C) asymmetric bias. 10.17630/779141ce-c26a-483b-bfee-4f12cf71d7b2. (TIF) [file pbio.3003094.s001.tif]

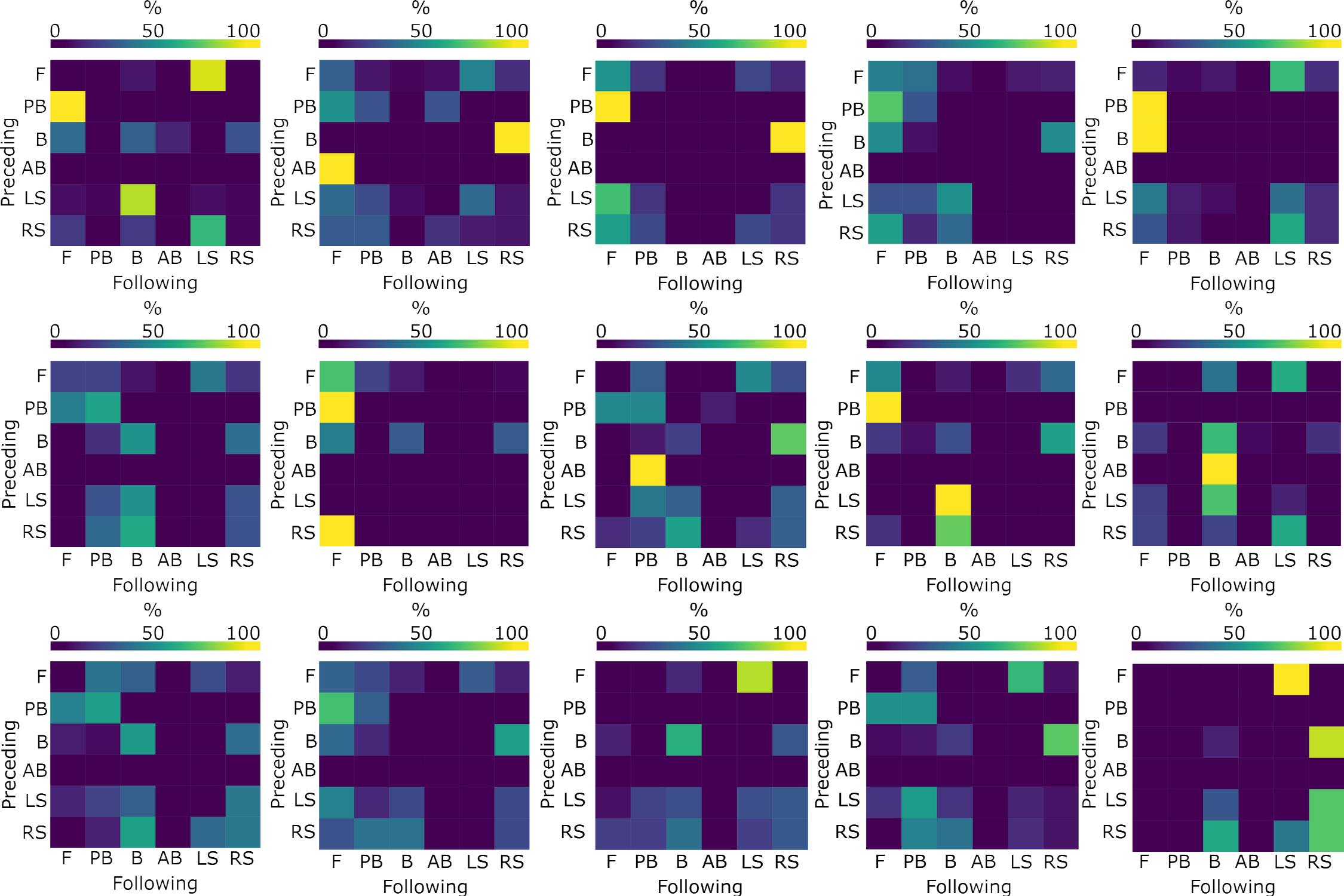

Supplement: S2 Fig — 10.17630/779141ce-c26a-483b-bfee-4f12cf71d7b2. (TIF) [file pbio.3003094.s002.tif]

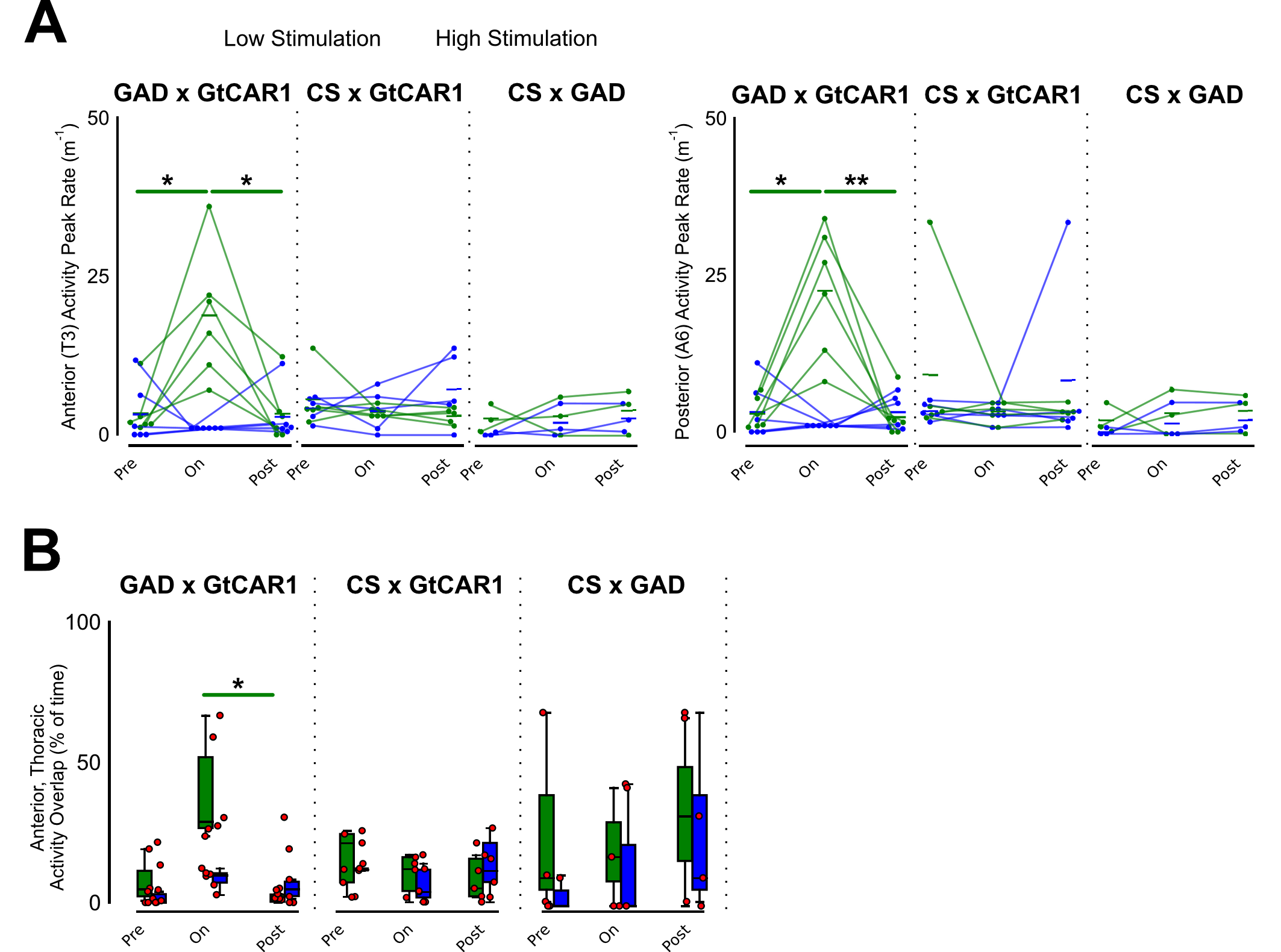

Supplement: S3 Fig — (A) Count per minute activity in anterior (T3) and posterior (A4-6) segments in GAD x GtCAR1 and control CS x GtCAR1 preparations. (B) Percentage of overlap between the anterior and posterior activity during the 60s stimulation period. Note, disinhibition at low optogenetic stimulation (green, 0.012 µ W/cm2) induces increased activity rate with more overlap between anterior and posterior segments whereas disinhibition at higher optogenetic stimulation (blue, 0.27 µ W/cm2) collapses rhythmic activity across segments. 10.17630/779141ce-c26a-483b-bfee-4f12cf71d7b2. (TIF) [file pbio.3003094.s003.tif]
