# Supplementary material for: Inhibitory circuit motifs in Drosophila larvae generate motor program diversity and variability
Source: PLoS Biol. 2025 Apr 21;23(4):e3003094. doi: 10.1371/journal.pbio.3003094 (PMC12088524; doi:10.1371/journal.pbio.3003094)
Supplement: Attachment — Submitted filename: Francis et al responses to reviewers FINAL.docx [file pbio.3003094.s006.docx]

**Francis et al. submission to PloS Biology**

**We thank the reviewers for their helpful and constructive comments. Below is a point-by-point response to their queries, with our responses *italicized and in bold***

Reviewer #1: Generation of motor program diversity and variability through inhibitory circuit motifs in the Drosophila larval locomotor system

Francis et al. submitted to PLoS Biology.

The authors generate a computational model that can recapitulate several features of the neural activity observed in isolated nerve cords of the Drosophila larval CNS.

    	The first place where this paper departs from earlier work (e.g., Pulver et al., Jonaitis et al. 2022) is a much more detailed examination of fictive motor patterns (Figures 1-5) using calcium imaging in CNSs isolated from Drosophila larvae. These data confirm and extend prior observations, with, in my opinion, an interesting and important new treatment of "anterior bursts" and "posterior bursts" and a more nuanced understanding of axial diversity in wave propagation. These descriptions later go on to inform many of the modeling decisions. A major strength of this paper is the data-informed approach.

    	Similar to previous models (Gjorgjieva et al., 2013), this paper's model comprises chains of local excitatory and inhibitory oscillators that can propagate wave-like activity along the axis. Here, the details of the oscillators differ slightly, and likely importantly, from prior work. However, this is not fully discussed. More novel is the incorporation of a second layer of "surrounding architecture," including neurons that detect the initiation of a wave and neurons activated for the wave's duration.

    	This work is extremely significant to the field of Drosophila larval behavior. First, it summarizes a diversity of work into a simple, powerful, plausible model. Second, this summary is useful for predicting the logical architecture of locomotor circuitry. More broadly, this work will interest any reader interested in the neural basis of behavior and those interested in computational modeling, physiology, and connectomics.

    	The claims are properly placed in the context of previous literature. One small note is that if they cite Vaddia et al., 2019, they should also cite He et al., 2019, as those papers came out back to back in the same journal.

The data and analyses support the major claims. In my opinion, no further work is needed. One note is that several graphs' text and other visual elements are too small to read without additional magnification.

    	I found this paper to be well-written and enjoyable to read.

***We have now included He et al., 2019, Current Biol. in the references.***

***We have also reviewed all figures a and have increased font size of several graphs’ text (specifically in Fig 1 A, Fig. 3A, Fig. 7A, Fig.8C).***

Reviewer #2: In this study, Francis et al. build a circuit-based model capable of recapitulating the diversity and variability of motor programs observed in isolated CNS preparations of Drosophila larva.

The authors first generate experimental data (Calcium imaging of fictive motor programs in isolated CNS) that they use, along with data from previously published studies to analyze the patterns of motor activity in these preparations. They characterize the type of motor programs they generate, their frequency as well as diversity within and between preparations.

They further build a model capable of generating rhythmic activity. The model is based of incorporating principles of circuit organisation in the larval motor system (based on published connectomics data) without including the full connectivity matrix of such a network. They build a minimal circuit module composed of two excitatory and an inhibitory neuron that is repeated across segmentz and incorporate elements (like for example wave detectors and command neuron) that allow to reproduce the features of the motor pattern activity observed in the larval CNS. They specifically explore the role of inhibitory motifs in generating oscillations and show (both experimentally and in the model) that inhibition in the circuit is essential for generating rhythmic activity and the variability of motor programs while in the same time preventing temporal overlap of the different activity patterns.

The logic for model building is sound and the simplified network is able to reproduce the basic feature of motor activity. As such, the model provides the basis upon which more complete models could be build, like those incorporating proprioceptive and exteroceptive inputs and that could reproduce other types of motor programs of other types of sequence of motor activity, as they describe in the discussion. Such models could generate testable predictions about how network activity generates motor programs and specifically transitions between motor programs in different context and could be of interest to a wide range of neuroscientists and specifically those studying motor systems, both theoreticians and experimentalists.

I onlly have minor suggestions for the authors:

The paper is overall well written and clear. Some sections of the results are sometimes overly descriptive. Streamlining those would, I believe, make the paper easier to read. Details could be kept in figure legends and figures.

For example : l199-l208, l525-l548, …

*W****e have reviewed the results section and have streamlined three sections (l199-l208, l525-l548, and L463-476) to make the paper easier to read.***

Line 802 -hunching, should be defined (i.e. head retraction, a defensive actions…)

***Hunching is a behaviour characterized by head retraction and simultaneous,  bilaterally symmetric  muscle contractions across multiple body segments. This is typically associated with responses to mechanical stimulation. In the sentence, we mention feeding, hunching, rolling. We have now provided a short description of hunching and a relevant reference for each behaviour.***

In some figure panels, the font is too small to read (e.g. Fig 1 A, Fig. 3A, Fig. 7A, Fig.8C)

***As noted above, we have reviewed all figures a and have increased font size of several graphs’ text and visual features (specifically in Fig 1 A, Fig. 3A, Fig. 7A, Fig.8C).***

Reviewer #3: The manuscript by Francis et al. describes the involvement of inhibitory circuit motifs in the diversity and variability observed in locomotion in fruit fly larvae. The authors conducted calcium imaging of glutamatergic neurons in the isolated central nervous system and analyzed the statistics and transitions of motor programs that appeared spontaneously. They observed that a posterior burst resets the generation rhythm of forward waves. The authors examined the motor programs generated in reduced preparations containing the A6 to A8 segments and found a diversity in motor programs. They found that rhythmic activity induced by a muscarinic agonist depends on the activity of GAD-GAL4 positive neurons. Based on these observations, the authors developed a computational model partially inspired by interneurons characterized previously in this system. Individual segments are modeled by one inhibitor unit (referring to GDL neurons) and two excitor units. One excitor

(referring to A27h neurons) is for forward waves and the other is for backward waves (referring to A18b neurons). The model contains other components, such as feed-forward intersegmental excitation, command neurons for forward and backward wave initiation, initiation detector to avoid concurrent appearance of forward and backward waves, and wave detectors to prevent multiple waves at the same time. The authors observe several phenomena in the model mimicking larval motor patterns: Waves are interrupted by blocking excitors or activating inhibitors. When stochastic inputs are applied to command neurons, transitions of motor programs are observed. By incorporating thoracic circuits, the authors built a fully integrated circuit model. This model generates diversity and variability resembling activity patterns observed experimentally in the isolated CNS. The effect of distinct levels of disinhibition is similar between optogenetic experiments and the computer model. The authors

show that the loss of function of different inhibitory motifs leads to distinct phenotypes in the computational model.

The physiological experiments and computer simulations are well-designed and properly executed. The theme that the authors are trying to address is quite important in neuroscience and could attract a wide range of researchers in both experiment and theoretical neuroscientists. However, there are several critical concerns regarding the manuscript that the authors need to clarify.

Major comments:

Page 15: GAD-Gal4 is used to express GtAcR1 in GABAergic neurons. However, the expression pattern of this driver is not cited or shown. Please clarify this.

***Apologies for this oversight. We used a GAD1-GAL4 driver created jointly by Ben White and Matthias Landgraf and published in (Diao et al., 2016, Cell Reports) and subsequently validated and used as driver for GABAergic neurons in follow on publications. Further details on this construct and our electrophysiological recordings are now presented in methods and materials.***

Page 16: The authors state about hemisegments but the hemi-cord preparation is not used. This should be clarified.

***It is technically unfeasible to  isolate a single hemi-segment, especially in the posterior regions of this preparation. Slicing midline connections without damaging hemi-segments is just too unpredictable as the larval VNC tapers to a point in posterior regions. In previous work, however, we have indeed experimented with hemi-chord preparations and showed that rhythmic activity can persist in more anterior abdominal hemi-segments when midline connections are severed, providing evidence for independent oscillators in hemi-segments (Jonaitis, 2022, J. Neurophysiol.). We felt that it was not worth re-doing those results here. Instead, we wanted to present new data showing that hemi-segments in preparations with small numbers of intact posterior segments are not all running on a single ‘clock’ but rather capable of oscillating at different frequencies simultaneously.  This, combined with our previous findings  provides a constellation of evidence arguing for the presence of independent oscillators in hemi-segments.***

Page 19: The hemisegmental inhibitory ("the inhibitor") is modelled referring to GDL. Although Fushiki et al. shows the necessity of GDL in forward waves, there is no evidence that shows GDL is required for backward waves. Some other evidence or observations supporting the existence of inhibitory interneurons modelled by "the inhibitor" should be described.

***The abstract of the Fushiki paper we cite*** [***https://pmc.ncbi.nlm.nih.gov/articles/PMC4829418/***](https://pmc.ncbi.nlm.nih.gov/articles/PMC4829418/) ***states that GDL is necessary for both forward and backward locomotion (line 8). This is also explicitly stated in paragraph 4 of the Introduction. In Figure 2 of Fushiki et al., GDLs are shown to be jointly recruited into both forward and backwards waves. Following figures show that inhibiting activity in GDLs does indeed impede forward wave activity. However, the reviewer is correct in saying that the authors do not present much evidence to demonstrate that GDL neurons are strictly required for backwards locomotion. In our manuscript here, however, we do show that strong optogenetic inhibition of GABAergic neurons completely collapses rhythmic activity underpinning both forward and backward waves in isolated preparations, suggesting that activity of GABAergic neurons is critical for both types of motor programmes. So overall, GDL neurons are implicated in wave-like activity in this system and are recruited into both forward and backward motor programmes, all of which is consistent with our model.***

***Importantly, however, our model is meant to be a distillation and simplification of the actual circuit, and the inhibitor in the model is not necessarily meant to be an exact replica of GDL per se. Rather it is meant to represent the concept of shared segmental inhibitory neurons being at heart of rhythm generation as proposed in Gjorgjieva et al. (2013). We feel that at this stage, this concept is sound, and well supported by existing evidence. But of course, the concept should be subject to further investigation in future work. We provide testable predictions and ideas for future experiments in the section of the Discussion where we discuss the Hemisegmental oscillator.***

***Overall, we appreciate the importance of accuracy and precision and delicacy on this point, and we have now reworded the sections where we mention GDL and have removed statements about those neurons being strictly required for backward locomotion.***

Page 20: The model includes feed-forward excitation based on Kohsaka et al. (2019). However, Kohsaka et al. (2019) doesn't report feed-forward motifs but feedback motifs. Biological evidence or observations supporting the intersegmental connection built in the model are required.

***Apologies for the miscue. We have now cited Hasegawa et al. (2016)*** [***https://pubmed.ncbi.nlm.nih.gov/27470675/***](https://pubmed.ncbi.nlm.nih.gov/27470675/) ***and Zarin et al. (2019).*** [***https://pmc.ncbi.nlm.nih.gov/articles/PMC6994239/***](https://pmc.ncbi.nlm.nih.gov/articles/PMC6994239/#fig9s1I) ***both of which show evidence for this type of feedforward connection being present.***

Page 24-27: The authors show that the model inspired by the Drosophila larval CNS combined with noise according to Poisson distribution exhibits diversity and variability. However, the involvement of inhibitory circuit motifs in diversity and variability in Drosophila larval locomotor system, as described in the title, is not shown. The data supporting the title statement is necessary.

***The inhibitory motifs in our model enable diverse and variable motor activity while also preventing maladaptive overlap of motor programmes, so we feel the title is appropriate. We focus on the functional roles of inhibitory motifs throughout the results and show how removing these motifs shapes diversity and variability. We then discuss links between inhibitory motifs and diversity and variability in a wider context in the discussion. We agree though that we could have been more explicit in linking up ideas and we have now more explicitly commented on how our results provide evidence for our title in the closing sections of the results and in the discussion.***

Page 37: There are three issues on Figure 14: 1) The traces in Figure 14Ai look quite different from Figure 14Bi and 14Ci. Please explain this.

***This is just a reflection of the high variability and diversity possible from preparation-to-preparation, and indeed moment-to-moment in individual preparations. We now present the pooled data from these experiments in Supplemental Figure 3, so that readers can see and understand the variability across experimental and control genotypes.***

2) There is no effector control (CS>GtAcR1) to check the effect of the leaky expression of UAS-GtAcR1.

***We now present data from C>GtACR1 experiments showing that continuous light pulses do not trigger any responses (and therefore that leaky expression of the UAS line is not an issue). To keep the figure compact, we would like to still only show one of the controls in the main figure; however, our new supplemental figure will allow readers to see pooled data from relevant controls.***

3) Quantitative analysis of the effect of blocking GABAergic neurons is missing. Readers cannot interpret how "representative" the data shown in Figure 14 are in the group of n = 4 preparations.

***We now present pooled data from our experimental preparations and have done additional experiments to increase sample size in experimental and control preparations. At low light intensities there is an increase in burst frequency and also an increase in overlap between activity patterns. At high intensities there is a single burst of motor activity followed by a complete collapse of rhythmic activity.***

Page 41 and 42: The ability of the muscarinic receptor agonist to induce forward waves, which is reported in Jonaitis et al., 2022, is cited several times. However, how this finding is reflected in the model is not clear. Please describe this.

***In Jonaitis et al. (2022) we showed that signalling through excitatory muscarinic receptors (mostly likely A-type receptors) was critical for rhythm generation underlying both forward and backward waves as well as fictive headsweeps in our system. We also showed that increasing mAChR signalling led to broad scale increases in tonic depolarization (and vice versa). Rhythm generation appears therefore to be conditional upon tonic excitation through mAChRs. Conceptually, this is represented in our model by the conditional nature of the Hemi-Segmental Oscillator. In our model, in order for oscillations to begin,  a tonic ‘kick’ is needed from an excitor, which in the actual animal appears to be mediated through mAChR signalling.***

***We have now reworded some sentences to better explain our reasoning on this point.***
